# The Correlation Between Maxillary Central Incisor Dimensions and Different Points on the Face in a Syrian Population

**DOI:** 10.1155/2024/5980986

**Published:** 2024-10-16

**Authors:** Yosha Ammar, Rima Saker

**Affiliations:** Department of Fixed Prosthodontics, Faculty of Dentistry, Tishreen University, Latakia, Syria

**Keywords:** anthropometry, esthetic dentistry, facial dimensions, MCI dimensions, Syrian

## Abstract

**Statement of the Problem:** When replacing the maxillary central incisor (MCI) or adjusting its dimensions, Syrian dentists may have difficulties in selecting the appropriate size due to the lack of a dental anthropometric database for Syrian society.

**Purpose:** The purpose of this observational study was to investigate the correlation between MCI dimensions and face length and width in Syrian females and males.

**Materials and Methods:** The study included 180 Syrian participants (90 females and 90 males) without facial or dental defects and aged between 20 and 25 years. A digital photograph of each individual's face was taken to measure face length and width. Maxillary stone casts were made for each individual to assess the MCI width and height using a digital caliper. The correlation between facial and dental variables was investigated with the Pearson correlation coefficient. Moreover, intersex variations were tested using an independent-sample *t*-test (*p* < 0.05 considered significant).

**Results:** The mean MCI width was 8.58 mm, the mean MCI cervical width was 7.96 mm, the mean MCI height was 9.69 mm, the average MCI width-to-height ratio was 89%, and the average MCI cervical width-to-height ratio was 82%. The intersex variations in dental values were not statistically significant. None of the studied facial dimensions showed any significant correlation with any of the MCI measurements.

**Conclusion:** The faces of Syrian men were larger than those of Syrian women, but their MCIs had similar proportions and sizes. Facial height and width were not the appropriate parameters for determining MCI size for Syrians.

## 1. Introduction

Credit for founding the modern craniofacial anthropometry is given to the scientist Leslie Farkas. Farkas pointed out that linear measurements alone are not sufficient, but they are necessary to study the relationship between measurements (craniofacial proportions) to evaluate the craniofacial complex [1]. The proportional and angular analysis was used in the planning of dental treatment, especially in orthodontics and cosmetic dentistry [2]. The maxillary central incisor (MCI) plays an essential role in the appearance of the smile [3]. The proportional relationship between MCI dimensions and face measurements has been edited out to select the most appropriate MCI for each face, and on its basis, we choose the rest of the teeth [4].

A variety of facial measurements were used to determine the MCI dimensions including bizygomatic width (BW), bigonial width, intercommisural width, interalar width, interpupillary distance, and intercanthal distance in order to predict the maxillary central incisor width (MCI W) [3, 5–8]. Furthermore, different facial lengths such as total face height, facial height (FH), and lips thickness were used to indicate the maxillary central incisor length (MCI L) [5, 8, 9].

One of the oldest dental–facial ratios is the 1:16 ratio of MCI L to FH as an ideal proportion [9]. Berry suggested the same rate, but between the mesial–distal MCI W and the BW [10]. The 1:16 MCI W-to-BW ratio was observed by Hasanreisoglu et al. among the female group only [3], while Radia et al. found it to be 1:15.5 [5]. Moreover, they proposed the 1:18 MCI L to total FH [5]. Researchers suggested the proportion between dental and facial variables based on arithmetic mean. However, Pearson correlation coefficients (PCCs) showed a poor correlation [5].

Ethnicity is a vital influence factor on dental anthropometric values [11]. MCI was significantly wider in African American than in Asian or White Americans [12, 13]. Additionally, the Kenyan teeth were larger than Irish teeth [14]. In another study by Bishara et al. [15], they observed significant differences in dental measurements among Americans, Egyptians, and Mexicans. As a result, the normative data of an ethnic group could not be applied to others [1].

Gender is considered another dominant influence factor. Several studies demonstrated that MCI is significantly wider and longer in males than in females [5, 8, 11], while the MCI W/L was less affected by sex [11]. Others did not find intersex variations [16]. A study conducted on the dimensions of upper anterior teeth among Kurds and Arabs found that the MCI W/L was the same for Arab males and females, but it was larger for Kurdish females than for males [17].

The current research has several fundamental goals: firstly, to examine the possibility of using some facial dimensions as a reference when selecting anterior teeth and, secondly, to compare the dimensions of MCI between Syrian men and women.

The null hypothesis was that, among Syrians, there is no correlation between facial dimensions and MCI W or maxillary central incisor height (MCI H). Additionally, there are no gender differences.

## 2. Materials and Methods

The protocol of the study had been approved by the Ethics Committee of the College of Dentistry Research Centre at Tishreen University under approval [2058] during session [14], held on April 5, 2022.

The present study used a cross-sectional descriptive design. It took place from January to August 2023 at Tishreen University. A total of 180 subjects (90 women, 90 men) were recruited for the study using a purposive nonrandomized sampling technique. In this research, horizontal and vertical facial measurements were obtained along with the width, cervical width, and height of the MCI. The subjects were included after the necessary ethics committee approvals were obtained. The participants aged between 20 and 25 years. The minimum age was set at 20 to ensure the complete maturation of the craniofacial complex and gingival tissue [18, 19], while the maximum age was set at 25 to reduce the likelihood of periodontal disease and tooth wear [20]. All participants were Syrian and had normal complete MCIs and healthy periodontium. The subjects who had restorations, caries, deformity or fractures on the MCIs, gingival swelling or recession, congenital or acquired defects in the head region, and facial surgery and bearded men were not included in this study.

**Figure 1:**
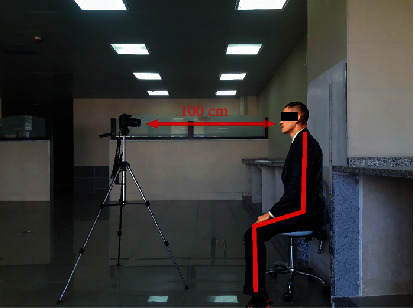
The individual in NHP, 100 cm from the camera.

Clinical measurements were performed at the level of the face on the one hand and the right MCI on the other hand. Facial measurements were carried out using AutoCAD software 2020 (AutoDesk, San Francisco, California) after taking digital photographs of the subjects. The photography conditions were uniform for all subjects. The photographs were made with a Canon 1200D mounted on a tripod which was adjusted according to each subject. The individual sat in the natural head position (NHP) and with lips at rest and teeth in central occlusion. Standardized photographs were taken from a distance of 100 cm and with white background and an opaque white ruler close to the individual, making sure that the person and the ruler are perfectly focused to ensure the accuracy of the enlargement (see Figure 1).

After calibrating the images to obtain life-size photographs, two trained researchers evaluated the images individually by using AutoCAD. The coordinates of soft tissue points in the *x* and *y* planes were extracted, and the distance between each landmark marked by each examiner was measured. Based on reading differences, landmarks were classified [21]. Readings that differed by less than 0.5 mm were highly reproducible, those that differed by more than 0.5 mm, but not by more than 1 mm, were moderately reproducible, and those that differed by more than 1 mm were poorly reproducible. A kappa coefficient test was used to assess interexaminer calibration for variables. The target level was ≥ 75% to ensure good agreement among the examiners.

**Figure 2:**
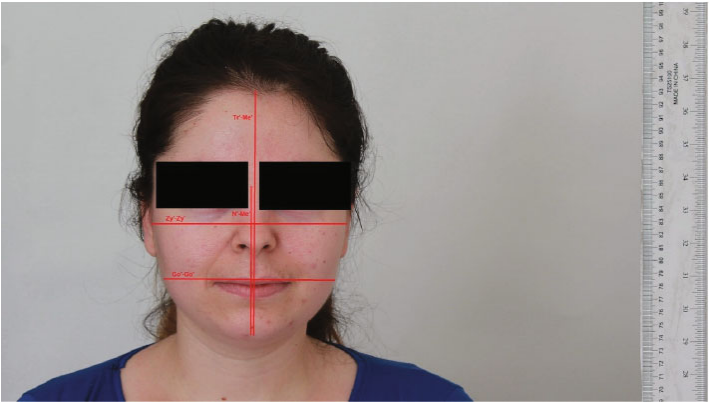
Vertical and horizontal facial measurements (Tr⁣′-Me⁣′, N⁣′-Me⁣′, Zy⁣′-Zy⁣′, and Go⁣′-Go⁣′).

A total of four dimensions were measured on the face, including two verticals (total face height/Tr⁣′-Me⁣′/; FH/N⁣′-Me⁣′/) in addition to two horizontals (BW/Zy⁣′-Zy⁣′/; mandibular width/Go⁣′-Go⁣′/) (see Figure 2) (see Table 1).

Dental measurements were accomplished on the casts using a digital caliper (Digital Vernier Caliper 80756, Draper, United Kingdom). In order to prepare the dental casts, irreversible hydrocolloid imprints (fast setting alginate hydrogum, Zhermack, Italy) were obtained from the maxillary arch. It was manipulated according to the manufacturer's instructions. After removing the impression from the patient's mouth, it was washed under running water in order to get rid of debris. For disinfection, all impressions were immersed in dimethyl ammonium chloride solution. Casting was performed immediately using dental stone (Elite Rock, Zhermack, Italy). After 30 min, the dental casts were extracted from the impressions. Two trained researchers carried out all the dental measurements. The measurements were compared for each case separately to ensure the work accuracy. If the difference was more than 0.2 mm, the measurement was repeated, while if the difference did not exceed 0.2 mm, the arithmetic mean of the values was taken.

**Figure 3:**
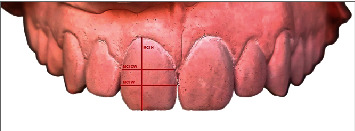
Vertical and horizontal dental measurements (MCI L, MCI W, and MCI CW).

Studied dental dimensions included the MCI H which was measured from the most apical point on the gingival margin to the incisal edge, in addition to the MCI W on the widest mesiodistal portion (MCI W) and on the cervical third (MCI CW) (see Figure 3).

## 3. Results

The descriptive statistics (mean, standard deviation, range, and 95% confidence intervals) of the recorded measurements are given in Table 2.

Looking at the right MCI dimensions, the mean MCI H for the women group was less than that reported for men (9.64 vs. 9.75 mm) in addition to MCI W (8.54 vs. 8.63 mm); the difference was not statistically significant, while the mean MCI cervical width was nearly equal (7.97 vs. 7.95 mm). Moreover, the MCI W/H ratio ranged between 0.827 and 0.947, and the MCI CW/H ratio was 0.827 ± 0.06, with no intersex differences.

From the descriptive facial measurements, Syrian women had statistically significantly lower facial dimensions than Syrian men (Tr⁣′-Me⁣′, 181.25 vs. 186.21 mm; N⁣′-Me⁣′,125.47 vs. 130.33 mm; Zy⁣′-Zy⁣′, 134.48 vs. 139.98 mm; and Go⁣′-Go⁣′ width, 114.45 vs. 121.80 mm). A minimum variation was noticed in Zy⁣′-Zy⁣′/Tr⁣′-Me⁣′ and Zy⁣′-Zy⁣′/N⁣′-Me⁣′ between males and females with no clinical significance.

PCC analysis of the relationship between the tooth variables and the face variables revealed that there was no statistically significant linear correlation, regardless of the gender of the individual (see Table 3).

## 4. Discussion

Since dental facial measurements are influenced by ethnic and societal differences [1], Syrian society-level measurements were necessary. Dentists could use the results found in this study to select teeth with ideal dimensions when planning a dental treatment.

In relation to MCI W, the results of the current study are consistent with studies conducted on Arabs [22, 23], Whites [5, 12, 24], and Asians [12, 25]. As compared to MCI W values on Africans [12, 26], Syrians' MCIs are narrower than Africans' (see Table 4).

The MCI L for Syrians was close to the results obtained on other societies [6, 22, 24, 26, 27]. However, it was shorter compared to studies conducted on Whites by Radia et al. [5] (see Table 4).

The MCI CW has been studied less compared to MCI W. In our study, the mean MCI CW was 7.96 mm (± 0.52 mm). This result was less than that reported by Ahmed et al. [28] (8.354 ± 0.487 mm) and greater than that reported by Yin et al. [27] (7.51 ± 0.62 mm). In addition to the variation in ethnic background, the difference here may also be attributed to the measurement methods, since they used 3D digital models versus our own dental casts.

The dimensions of MCI did not vary by gender in this study, comparable to Sayed et al.'s [6] study on Arabs, but contrary to the studies carried out by Chunhabundit et al. [8], Radia et al. [5], and Wang et al. [11].

The shape of MCI is fundamentally influenced by the W/L ratio; as the ratio increases, the MCI tends to take a square shape, while as it decreases, it takes an elongated shape. The W/L ratio in our study exceeded the esthetic value (0.75–0.78) proposed by Rosenstiel et al. [29] and was far from the golden ratio (1:1.1618). Similar to studies reported by Sayed et al. [6] and Al-Kaisy and Garib [17] on Arabs, we found that the W/L ratio was not affected by gender, in contrast to the Kurdish population. The MCI W/L in the Syrian population was 0.887, agreeing with Al-Kaisy on Arabs, but it was less in the Kurdish population, thus further supporting the belief of the racial difference of dentofacial proportion.

Syrian men had significantly wider and longer faces than women which is in agreement with other populations [5, 12]. As compared to studies conducted by Ouni et al. [37] and Sayed et al. [6] on Arabs, we found higher facial measurements (see Tables 5 and 6). The discrepancy in the results may be because they measured directly on the face, which may result in lower values due to the compressibility of the soft tissues. In contrast, Ahmed, Al-Labban, and Nahidh's [38] results matched ours, who used similar measurement methods.

This study examined MCI proportions (W/L, CW/L) and some facial proportions (Zy⁣′-Zy⁣′/Tr⁣′-Me⁣′, Zy⁣′-Zy⁣′/N⁣′-Me⁣′) and found that neither MCI W/L nor face W/L were affected by gender; we also investigated the relationship between the two variables without finding any significant results. The same conclusion was reported by Chunhabundit et al. [8].

In order to select the most suitable prosthetic frontal teeth for each face, facial measurements are often used. A variety of studies have been published in this area, with varying results depending on measurement methods and ethnic background. According to Berry [10] and House and Loop [9], the ratio of the MCI W to Zy⁣′-Zy⁣′ is 1.16. Furthermore, MCI L has been proposed to be 1:18 as compared with total FH [5]. This study revealed a ratio of 1:15.8 for MCI W to Zy⁣′-Zy⁣′ and 1:19 for MCI L to Tr⁣′-Me⁣′.

None of the studied facial dimensions showed any significant correlation with any of the MCI measurements. This result is in agreement with Sayed et al. [6] and Chunhabundit et al. [8]. Because of that, we cannot rely on Zy⁣′-Zy⁣′, Go⁣′-Go⁣′, N⁣′-Me⁣′, or Tr⁣′-Me⁣′ to indicate the MCI W or MCI H for the Syrian population.

This study confirms that anthropometric measurements differ according to race. Our results on Syrian society are closer to those of other studies on Arabs than those on other societies.

The present study has some limitations. (1) This study used a larger sample size than many comparable studies to explore the relationship between face dimensions and MCI dimensions. However, our sample size is still relatively small to be able to evaluate the normal values of the facial and dental anthropometric measurements. In order to validate our findings, further studies with larger numbers of participants are necessary. (2) Some landmarks were difficult to identify in 2D photos. Therefore, future studies should consider utilizing 3D models to measure facial dimensions. (3) We did not study some confounding variables like head type (dolicho-, brachy-, and mesocephalic) which may affect the findings of the study. (4) This study did not examine some facial variables whose relationship with MCI dimensions has been proven by other studies in other populations, since broader studies are needed. Despite these limitations, our study is the first of its kind in Syrian society, considering it a cornerstone to carry out broader studies.

## 5. Conclusions

The result of this study revealed the following outcomes:

1. Syrian men's faces were wider and longer than Syrian women's faces.
2. There were no significant differences between Syrian males and females in MCI dimensions.
3. Face and MCI proportions were less affected by gender.
4. Since the investigated facial dimensions (Zy⁣′-Zy⁣′, Go⁣′-Go⁣′, N⁣′-Me⁣′, and Tr⁣′-Me⁣′) did not correlate with MCI W and MCI L, the use of facial width or length as dependent parameters to estimate the dimensions of MCIs in the Syrian population was not convenient.
5. More studies are needed to investigate the relationship between the MCI size and other facial parameters for the Syrian population.

## Figures and Tables

**Table 1:** Facial soft tissue landmarks summary.

**Soft tissue landmarks pointed in this article**	
Trichion (Tr)	Midline point at the junction of the hairline and forehead
Nasion (N)	The midpoint on the soft tissue contour of the base of the nasal root at the level of the frontonasal structure
Menton (Me)	Most inferior midline point of the soft tissue chin
Zygion (Zy)	The most lateral point on the soft tissue contour of each zygomatic arch
Gonion (Go)	The most lateral point on the soft tissue contour of each mandibular angle

**Table 2:** Descriptive statistics for dental and facial parameters for both sexes: mean, standard deviation, and range. Comparison of variables between genders: M-F, *t* value, and *p* value.

**Variable (mm)**	**All (** **n** = 180**)**		**Females (** **n** = 90**)**		**Males (** **n** = 90**)**		**Male:female ratio**		
	**Mean (SD)**	**Range**	**Mean (SD)**	**Range**	**Mean (SD)**	**Range**	**M-F**	**t**	**p**
Tr⁣′-Me⁣′	183.73 (14.57)	117.3–220	181.25 (11.81)	152.3–207.6	186.21 (16.59)	117.3–220	4.96	2.312	0.022
N⁣′-Me⁣′	127.90 (8.99)	106.7–159	125.47 (7.96)	106.7–142.3	130.33 (9.34)	113.6–159	4.86	3.760	0.001
Zy⁣′-Zy⁣′	137.23 (10.51)	106.7–162.4	134.48 (9.30)	106.6–157.1	139.98 (10.98)	119.3–162.4	5.50	3.628	0.001
Go⁣′-Go⁣′	118.87 (9.22)	84.3–159.2	114.45 (8.77)	84.3–147.8	121.80 (9.33)	100–159.2	7.35	4.160	0.001
Zy⁣′-Zy⁣′/Tr⁣′-Me⁣′	0.750 (0.05)	0.616–1.083	0.744 (0.04)	0.616–0.831	0.756 (0.06)	0.66–1.083	0.01	1.638	0.103
Zy⁣′-Zy⁣′/N⁣′-Me⁣′	1.070 (0.10)	0.118–1.4	1.076 (0.07)	0.91–1.4	1.064 (0.12)	0.118–1.214	−0.01	−0.780	0.436
MCI L	9.69 (0.68)	7.8–11.98	9.64 (0.62)	8.01–11.06	9.75 (0.73)	7.8–11.98	0.12	1.151	0.251
MCI W	8.58 (0.52)	7.33–9.9	8.54 (0.52)	7.33–9.66	8.63 (0.52)	7.53–9.9	0.09	1.188	0.236
MCI CW	7.96 (0.52)	6.64–9.32	7.97 (0.55)	6.65–9.32	7.95 (0.50)	6.64–9	−0.02	−0.287	0.775
MCI W/L	0.887 (0.06)	0.75–1.06	0.888 (0.05)	0.725–1.06	0.866 (0.06)	0.75–1.041	−0.003	−0.346	0.730
MCI CW/L	0.824 (0.06)	0.677–1.05	0.829 (0.06)	0.677–1.05	0.818 (0.06)	0.684–0.996	−0.012	−1.237	0.218

**Table 3:** Pearson correlation coefficient (PCC) results for face:tooth dimensions.

**Face**	**Tooth**	**All (** **n** = 180**)**		**Females (** **n** = 90**)**		**Males (** **n** = 90**)**	
		**CI**	**p**	**CI**	**p**	**CI**	**p**
Tr⁣′-Me⁣′	MCI L	0.055	0.463	−0.034	0.748	0.087	00.415
N⁣′-Me⁣′	MCI L	0.076	0.313	−0.129	0.225	0.188	0.076
Zy⁣′-Zy⁣′	MCI W	0.012	0.870	0.036	0.739	−0.051	0.631
Go⁣′-Go⁣′	MCI W	−0.033	0.658	0.024	0.821	−0.099	0.353
Zy⁣′-Zy⁣′	MCI CW	−0.059	0.429	0.035	0.744	−0.140	0.188
Go⁣′-Go⁣′	MCI CW	−0.010	0.889	−0.009	0.936	−0.011	0.921
Zy⁣′-Zy⁣′/Tr⁣′-Me⁣′	MCI W/L	0.016	0.831	0.121	0.256	−0.044	0.682
Zy⁣′-Zy⁣′/Tr⁣′-Me⁣′	MCI CW/L	0.052	0.486	0.110	0.300	0.035	0.740
Zy⁣′-Zy⁣′/N⁣′-Me⁣′	MCI W/L	0.048	0.525	−0.077	0.468	0.111	0.297
Zy⁣′-Zy⁣′/N⁣′-Me⁣′	MCI CW/L	0.027	0.824	−0.069	0.517	0.055	0.604

**Table 4:** Previous studies about MCI dimensions.

	**MCI L (mm)**			**MCI W (mm)**			
	**All**	**Female**	**Male**	**All**	**Female**	**Male**	
In the current study	9.69 (0.68)	9.64 (0.62)	9.75 (0.73)	8.58 (0.52)	8.54 (0.52)	8.63 (0.52)	Syrian

Aldegheishem et al. 2019 [22]/Saudi	9.728 (0.9115)	—	—	8.5246 (0.5611)	—	—	Arabs
Sayed et al. 2017 [6]/Saudi	—	9.22 (0.93)	9.67 (0.80)	—	—	—	
Al Wazzan 2001 [23]/Saudi	—	—	—	8.48 (0.55)	8.36 (0.56)	8.61 (0.5)	

Parciak et al. 2017 [12]	—	—	—	—	8.5 (0.57)	8.9 (0.48)	Asians
Hemalatha, Chander, and Anitha 2018 [25]/Indian	—	—	—	8.4 (0.96)	8.2 (0.81)	8.7 (1.11)	
Shetty, Beyuo, and Wilson 2017 [26]	9.55 (1.18)	—	—	8.79 (0.7)	—	—	
Yin et al. 2020 [27]	9.53 (0.77)	—	—	—	—	—	

Radia et al. 2016 [5]/British	—	10.06 (0.52)	10.52 (0.86)	—	8.54 (0.52)	8.8 (0.51)	Whites
Parciak et al. 2017 [12]	—	—	—	—	8.6 (0.5)	8.9 (0.51)	
Melo et al. 2019 [24]	—	9.1 (0.94)	9.59 (1.03)	—	8.44 (0.51)	8.73 (0.65)	

Parciak et al. 2017 [12]	—	—	—	—	9.1 (0.64)	9.4 (0.57)	Africans
Shetty, Beyuo, and Wilson 2017 [26]	—	9.5 (0.9)	9.7 (1.2)	—	8.8 (0.6)	9.1 (0.6)	

**Table 5:** Previous studies about facial width.

	**Go-Go (mm)**			**Zy-Zy (mm)**			
	**All**	**Female**	**Male**	**All**	**Female**	**Male**	
In the current study	115.65 (13.54)	112.34 (11.43)	119.87 (14.55)	137.23 (10.51)	134.48 (9.30)	139.98 (10.98)	Syrian

Sayed et al. 2017 [6]/Saudi	—	—	—	120.2 (13.96)	131.5 (8.80)	111.2 (10.32)	Arabs
Celebi et al. 2018 [30]/Egyptian	—	113.61 (6.54)	120.76 (6.65)	—	98.73 (6.45)	104.95 (7.31)	
El Minawi et al. 2022 [31]/Egyptian	—	103.94 (8.90)	—	—	119.21 (8.15)	—	
Farkas et al. 2005 [32]/Egyptian	—	91.2 (7.2)	97.9 (9.2)	—	130.3 (10.4)	139.8 (13.8)	

Alam et al. 2015 [33]/Malay	—	—	—	—	110.12 (15.15)	114.75 (10.10)	Asians
Alam et al. 2015 [33]/Chinese	—	—	—	—	115.19 (13.36)	117.10 (11.48)	
Alam et al. 2015 [33]/Indian	—	—	—	—	107.83 (13.76)	112.67 (9.63)	
Farkas et al. 2005 [32]/Japanese	—	115.6 (11.2)	117.3 (15.8)	—	141.2 (11.8)	147.2 (11.2)	

Radia et al. 2016 [5]/British	108.92 (8.20)	104.36 (6.78)	112.38 (7.56)	134.15 (6.89)	130.51 (4.89)	137.94 (6.64)	Whites
Celebi et al. 2018 [30]/Italian	—	114.28 (5.32)	123.59 (8.74)	—	98.29 (4.86)	105.57 (6.37)	
Farkas et al. 2005 [32]/German	—	91.5 (10.00)	97.6 (12.00)	—	123.4 (105.0)	133.2 (15.00)	

Virdi, Wertheim, and Naini 2019 [34]/Kenyan	—	96.8 (2.9)	106.6 (5.9)	—	130.1 (3.5)	133.8 (4.6)	Africans
Wilson, Medapati, and Segwapa 2023 [35]	—	—	—	—	139.2 (6.83)	—	
Soumboundou et al. 2023 [36]/Senegalese	—	—	—	—	117.9 (7.76)	122.0 (6.81)	
Farkas et al. 2005 [32]/Afro-American	—	96.7 (10.00)	104.2 (12.00)	—	130.5 (5.6)	138.7 (11.2)	

**Table 6:** Previous studies about facial height.

	**N-Me (mm)**			**Tr-Me (mm)**			
	**All**	**Female**	**Male**	**All**	**Female**	**Male**	
In the current study	113.65 (8.99)	109.44 (7.96)	117.23 (9.34)	183.73 (14.57)	181.25 (11.81)	186.21 (16.59)	Syrians

Sayed et al. 2017 [6]/Saudi	—	—	—	178.7 (9.41)	176.7 (8.31)	180.4 (9.93)	Arabs
Ouni et al. 2022 [37]	—	—	—	—	181.52 (10.30)	195.90 (11.22)	
El Minawi et al. 2022 [31]/Egyptian	—	117.10 (4.63)	—	—	183.88 (9.72)	—	
Farkas et al. 2005 [32]/Japanese	—	113.8 (11.4)	122.8 (13.8)	—	182.8 (14.6)	191.4 (16.6)	

Alam et al. 2015 [33]/Malay	—	—	—	—	161.83 (14.14)	179.05 (15.27)	Asians
Alam et al. 2015 [33]/Chinese	—	—	—	—	172.57 (22.52)	188.43 (14.00)	
Alam et al. 2015 [33]/Indian	—	—	—	—	168.28 (13.45)	178.25 (13.17)	
Farkas et al. 2005 [32]/Japanese	—	101.5 (11.00)	112.5 (11.4)	—	163.0 (17.4)	161.3 (4.6)	

Radia et al. 2016 [5]/British	121.16 (7.01)	117.40 (5.93)	125.08 (5.82)	183.15 (9.58)	178.19 (8.06)	188.34 (8.24)	Whites
Farkas et al. 2005 [32]/German	—	109.5 (10.00)	116.0 (17.4)	—	170.9 (14.4)	182.2 (22.2)	

Virdi, Wertheim, and Naini 2019 [34]/Kenyan	—	117.1 (5.4)	101.8 (3.6)	—	—	—	Africans
Soumboundou et al. 2023 [36]/Senegalese	—	107.1 (8.12)	111.7 (6.91)	—	—	—	
Farkas et al. 2005 [32]/Afro-American	—	116.5 (12.2)	125.9 (16.4)	—	180.1 (15.00)	194.6 (21.2)	

## Data Availability

The data used to support the findings of this study are included within the article.
